# PD-1 Blockade in Advanced Melanoma in Patients with Hepatitis C and/or HIV

**DOI:** 10.1155/2015/737389

**Published:** 2015-09-10

**Authors:** Diwakar Davar, Melissa Wilson, Chelsea Pruckner, John M. Kirkwood

**Affiliations:** Division of Hematology-Oncology, Department of Medicine, University of Pittsburgh Medical Center, Pittsburgh, PA 15232, USA

## Abstract

On the basis of remarkable antitumor activity, programmed death receptor-1 (PD-1) inhibitors pembrolizumab and nivolumab were approved for the treatment of advanced melanoma in the second-line setting following progression on either CTLA-4 inhibitor ipilimumab or BRAF/MEK inhibitors (for *BRAF* mutated melanoma). Given hypothesized risk of triggering exacerbations of autoimmune diseases and/or chronic viral infections, clinical trials (including regulatory studies) evaluating checkpoint blocking antibodies PD-1 and CTLA-4 have excluded patients with autoimmune diseases, chronic hepatitis B/C virus (HBV/HCV), and/or human immunodeficiency virus (HIV) infections. Herein, we describe two patients with advanced melanoma and concomitant HCV/HIV infections treated with PD-1 inhibitor pembrolizumab. Patient 2 with HIV/HCV coinfection progressed after 2 doses of pembrolizumab. Patient 1 who had HCV alone was treated with pembrolizumab with initial partial response. HCV viral load remained stable after 9 cycles of pembrolizumab following which 12-week course of HCV-directed therapy was commenced, resulting in prompt reduction of HCV viral load below detectable levels. Response is ongoing and HCV viral load remains undetectable. In both patients, no significant toxicities were observed when pembrolizumab was initiated. We argue for the further investigation of checkpoint inhibition in cancer patients with underlying chronic viral infections in the context of carefully designed clinical trials.

## 1. Background

Pembrolizumab is a programmed death receptor-1 (PD-1) blocking antibody approved for the treatment of metastatic melanoma that has progressed past cytotoxic T-lymphocyte antigen 4 (CTLA-4) inhibitor Ipilimumab and BRAF inhibitors such as vemurafenib or dabrafenib (if BRAF mutated). Pembrolizumab was granted accelerated approval by the Food and Drug Administration (FDA) on the basis of a phase I trial that evaluated two cohorts that received either 2 mg/kg or 10 mg/kg of pembrolizumab every 3 weeks in which investigators reported high response rates (38%–52%) with most of the responders (82%) remaining on treatment [1].

PD-1/PD-L1 and CTLA-4 play important roles in regulating the immune system; hence, patients with autoimmune diseases requiring systemic immunosuppression and/or patients with hepatitis B/C (HBV/HCV) or human immunodeficiency virus (HIV) infection have been excluded from studies evaluating these agents over concerns about inadvertent augmentation of infectious and/or inflammatory activity. Although anti-CTLA-4 treatment has been shown to trigger or worsen severity of autoimmune diseases in experimental models, a similar effect has not been shown for PD-1/PD-L1 abrogation [2–4].

We report on two patients with advanced melanoma and concomitant HCV/HIV infections (patient 1: HCV; patient 2: HCV and HIV) treated with PD-1 inhibition. In both cases, pembrolizumab was well tolerated with no exacerbation of underlying HCV/HIV infection or observed toxicity.

## 2. Case Presentation 1 (Patient 1)

A 59-year-old Caucasian female presented with a subcutaneous right breast lesion on screening mammography in August 2014. Ultrasound-guided biopsy revealed malignant cells with an immunophenotype consistent with metastatic melanoma. Physical examination was negative for a possible primary lesion. Molecular testing was negative for either* BRAF* V600 or* NRAS* codon 61 mutations. Staging positron emission tomography (PET) and magnetic resonance imaging (MRI) scans confirmed two metabolically active nodules in right lower lung with no evidence of metastases in other visceral structures, brain, or skeletal system.

Prior history was notable for HCV infection documented in March 2014 following mildly elevated blood alanine transaminase (ALT) and aspartate aminotransferase (AST) levels. HCV-specific characteristics included high viral load (2,290,867 IU/mL) and 1A genotype. Clinically relevant parameters included IL28B polymorphism CC genotype, mild-moderate active chronic hepatitis (Ishak index 6/18) with moderate portal/peri-portal hepatic fibrosis (fibrosis stages 2-3/6). Her social history was notable for history of intranasal cocaine and intravenous drug abuse between ages of 20 and 30. She was in a long-term monogamous relationship with her husband of 30 years without prior high-risk sexual partners.

**Figure 1:**
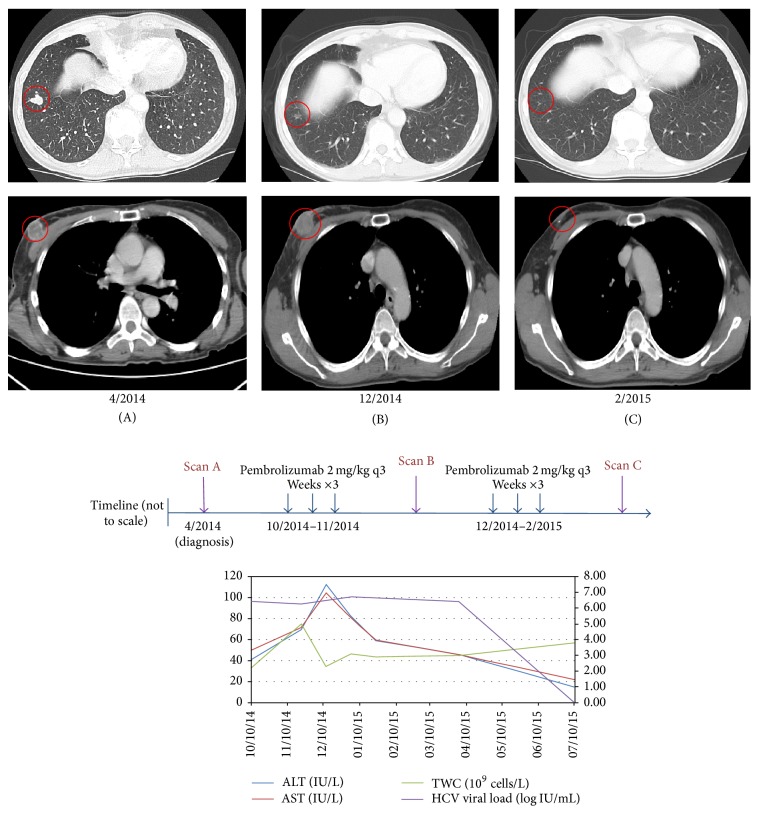
Changes in tumor size and correlation with laboratory results in patient 1. After 3 cycles, index right breast lesion (A-B, lower panel) increased in size while index right pulmonary lesion decreased in size consistent with immune-related response pattern (A-B, upper panel). After 6 cycles, both lesions had decreased in size significantly (C, upper and lower panels). Although pembrolizumab treatment was associated with grade 1 leucopenia and grade 1 ALT/AST elevations initially, total white count and ALT/AST levels subsequently stabilized (graph). HCV viral load fluctuated between 3.36 IU/mL (6.53 log IU/mL) and 5.17 IU/mL (6.71 log IU/mL) before initiation of ledipasvir and sofosbuvir in April 2015. After 12-week course, HCV viral load became undetectable and remains so. To date, she has completed 15 cycles to date with ongoing excellent partial response.

Given the minimal disease burden, we encouraged her to pursue initial HCV therapy followed by therapy for advanced melanoma given the recent approval of antiviral agents with unprecedented levels of antiviral activity in HCV. However, she elected against this. In the setting of mild-moderate hepatitis with moderate fibrosis and mildly elevated ALT/AST, we were concerned about a heightened risk of ipilimumab-related hepatitis. Following an extensive discussion of the available options and carefully considering the respective risks of treatment, she commenced therapy with PD-1 inhibitor pembrolizumab at 2 mg/kg every three weeks. Restaging scans following 3 cycles showed a mixed response, slight increase in size of right breast lesion and new hilar and right axillary lymphadenopathy although pulmonary lesions were significantly decreased in size with an overall reduction in total tumor burden. Restaging scans following further 3 cycles of therapy showed significant reduction in size of both hilar/axillary lymphadenopathy and pulmonary nodules consistent with partial response (see Figure 1). After 9 cycles of pembrolizumab with ongoing response, she commenced a 12-week course of ledipasvir (NS5A inhibitor) and sofosbuvir (viral RNA polymerase inhibitor). During cycle 1–3 of pembrolizumab, ALT/AST levels and HCV viral loads remained stable. Following commencement of ledipasvir/sofosbuvir (after 9 cycles of pembrolizumab), HCV viral load declined to below detectable levels. At the time of reporting, she has an ongoing excellent partial response after 15 cycles of therapy with pembrolizumab with normal ALT/AST and undetectable HCV viral load.

## 3. Case Presentation 2 (Patient 2)

A 47-year-old Caucasian male was diagnosed with a left axillary 0.8 mm nonulcerated melanoma in September 2001. Following negative wide excision and negative sentinel lymph node mapping, no adjuvant therapy was offered. He was followed closely with period examinations and presented with palpable left axillary lymphadenopathy in September 2012. CT imaging confirmed a 2.1 × 1.0 cm left axillary lymph node with no other evidence of metastases. Excisional biopsy of left axillary lymph node confirmed malignant melanoma and subsequently underwent completion lymph node dissection which revealed multiple foci of metastatic melanoma in 2 of 36 lymph nodes removed with no extracapsular extension. Molecular testing confirmed BRAF V600E mutation though NRAS codon 61 mutation was not identified.

Past medical history was significant for remote diagnosis HIV-1 and chronic HCV diagnosed in June 2011. He was treated with an antiretroviral therapy (ART) regimen that consisted of 2 nucleoside reverse transcriptase inhibitors and 1 nonnucleoside reverse transcriptase inhibitor. HIV-1 viral load was consistently undetectable. HCV-specific characteristics included low viral load (863,475 IU/mL) and 1C genotype. Clinically relevant parameters included IL28B polymorphism CT genotype and chronic hepatitis associated with mild activity (Ishak index 5/18) and mild-moderate portal/peri-portal fibrosis (fibrosis stage 2/6). His social history was notable for homosexual orientation with multiple prior high-risk partners though recently monogamous and there was no prior history of intravenous drug abuse.

He received adjuvant high-dose interferon (IFN) between December 2012 and May 2013, during which time HIV and HCV viral loads were unchanged. IFN was stopped given development of subcutaneous melanoma metastases. Given well-controlled HIV and HCV, he was offered CTLA-4 inhibitor ipilimumab and received 3 doses (3 mg/kg every 3 weeks) between October and December 2013, discontinued for progression. Subsequent treatments included 2 cycles of high-dose IL-2 (4 and 6 doses in cycles 1/2, resp.) from January to March 2014 and dabrafenib/trametinib from March to August 2014, discontinued for progressive cutaneous and pulmonary disease with new hepatic lesions. Given excellent performance status despite widely metastatic disease, PD-1 inhibitor pembrolizumab (2 mg/kg every three weeks) was initiated after careful consideration of risks and benefits of treatment and he received 2 doses. Throughout treatment, despite progressive disease, he experienced no immune-related adverse events (ir-AEs), substantial increases in HIV/hepatitis C viral loads, and/or any abnormalities in hepatic function studies. Throughout treatment, he was maintained on ART.

Unfortunately, following 2 doses of pembrolizumab, he developed brain metastases requiring stereotactic radiosurgery and declined in performance status. Pembrolizumab was stopped and he transitioned to hospice care. He passed away shortly thereafter.

## 4. Conclusions

We describe two patients with active HCV/HIV infections and advanced melanoma treated with pembrolizumab in the setting of limited alternative treatments. To our knowledge, this is the first report of advanced melanoma patients with active HCV or HIV infection to be treated with pembrolizumab. The PD-1/PD-L1 pathway is known to be upregulated in chronic viral (HBV, HCV, and HIV) infections where it may attenuate T-cell or NK-cell mediated antiviral host immune responses, thereby sustaining chronic infection [5]. Abrogation of PD-1/PD-L1 signaling may have benefit in chronic viral diseases and this strategy is being explored in both HIV-1 infection (NCT02028403) and HCC associated with HBV/HCV related hepatitis (NCT01658878) with anti-PD L1 BMS-936559 and anti-PD-1 BMS-936558, respectively. However, HIV/HCV patients have hitherto been systematically excluded from clinical trials of pembrolizumab in advanced melanoma and whether pembrolizumab truly exacerbates HCV/HIV infection remains unknown. Prior phase I studies of PD-1 inhibitor BMS-936558 and PD-L1 inhibitor BMS-936559 had similarly excluded patients with HCV/HIV infection [6, 7].

Although patient 2 did not demonstrate antitumor benefit from pembrolizumab (or any other prior treatment including the combination of dabrafenib/trametinib), HIV viral load remained undetectable likely secondary to continued compliance with ART therapy while on therapy. Previously received treatments including HD IL-2, CTLA-4 inhibitor ipilimumab, and PD-1 inhibitor pembrolizumab were discontinued secondary to clinical and/or radiologic progression rather than development of ir-AEs. Throughout treatment, HCV viral loads were variable though HIV viral load was undetectable. ALT/AST elevations were of no more than Common Terminology Criteria for Adverse Events (CTCAE) grade 1 severity.

Remarkably, patient 1 has demonstrated an ongoing sustained response to therapy following a mixed response at first staging evaluation. The clinical development of ipilimumab was notable for the heterogeneity of responses observed which led several authors to propose “immune-related response criteria” (irRC) to specifically evaluate responses to immunotherapeutic agents [8]. Using these criteria, physicians are permitted to continue therapy in the face of new lesions (measurable or nonmeasurable) as long as the overall tumor burden is stable or is declining. Ongoing PD-1/PD-L1 trials are evaluating response using RECIST v1.1 though response by irRC is often a secondary endpoint on these studies. Treatment with pembrolizumab was notable for variable pancytopenia and ALT/AST elevations of no more than CTCAE grade 1 severity. HCV viral load remained stable throughout therapy and became undetectable following initiation of anti-HCV therapy.

Given the incidence of chronic viral hepatitis (hepatitis B 0.4%; hepatitis C 1.0%) and HIV infection (0.4%) separately and HIV/HCV coinfection (up to 80% in intravenous drug abusers) and the rising incidence of melanoma, evaluating the safety of immunotherapies for the treatment of melanoma in these cohorts is of pressing importance. Additionally, viral hepatitis and HIV are global health scourges which have an outsized impact on patients in developing countries. Demographic and lifestyle changes in developing countries will likely result in cancer incidences that approach those of developed countries such as the United States, Australia, and the European Union. These trends coupled with the availability of highly active treatments that turn HCV and HIV into chronic illnesses make these diseases important considerations as we develop and evaluate effective antitumor immunotherapy.

Anecdotal reports are not substitutes for well-conducted clinical trials to evaluate the safety and efficacy of agents in specific settings. Moreover, our experience with these two patients is in no way generalizable to other patients. However, this experience argues for the systematic evaluation of PD-1/PD-L1 immunotherapy in patients with chronic HBV/HCV and/or HIV infection and potentially other entities including autoimmune illnesses requiring systemic immunosuppression, either as extensions of existing studies or within the confines of an organ dysfunction trial.

## Abbreviations

ALT: Alanine transaminase
ART: Antiretroviral therapy
AST: Aspartate aminotransferase
CTLA-4: Cytotoxic T-lymphocyte-associated protein 4
FDA: Food and Drug Administration
HBV: Hepatitis B virus
HCV: Hepatitis C virus
HIV: Human immunodeficiency virus
IFN: High-dose interferon
ir-AE: Immune-related adverse events
MRI: Magnetic resonance imaging
PD-1: Programmed death receptor-1
PET: Positron emission tomography.

## References

[1] Hamid O., Robert C., Daud A. (2013). Safety and tumor responses with lambrolizumab (anti-PD-1) in melanoma. *The New England Journal of Medicine*.

[2] Perrin P. J., Maldonado J. H., Davis T. A., June C. H., Racke M. K. (1996). CTLA-4 blockade enhances clinical disease and cytokine production during experimental allergic encephalomyelitis. *The Journal of Immunology*.

[3] Lühder F., Höglund P., Allison J. P., Benoist C., Mathis D. (1998). Cytotoxic T lymphocyte-associated antigen 4 (CTLA-4) regulates the unfolding of autoimmune diabetes. *Journal of Experimental Medicine*.

[4] Wang H.-B., Shi F.-D., Li H., Chambers B. J., Link H., Ljunggren H.-G. (2001). Anti-CTLA-4 antibody treatment triggers determinant spreading and enhances murine myasthenia gravis. *The Journal of Immunology*.

[5] Wang X. F., Lei Y., Chen M., Chen C. B., Ren H., Shi T. D. (2013). PD-1/PDL1 and CD28/CD80 pathways modulate natural killer T cell function to inhibit hepatitis B virus replication. *Journal of Viral Hepatitis*.

[6] Topalian S. L., Hodi F. S., Brahmer J. R. (2012). Safety, activity, and immune correlates of anti–PD-1 antibody in cancer. *The New England Journal of Medicine*.

[7] Brahmer J. R., Tykodi S. S., Chow L. Q. M. (2012). Safety and activity of anti-PD-L1 antibody in patients with advanced cancer. *The New England Journal of Medicine*.

[8] Wolchok J. D., Hoos A., O'Day S. (2009). Guidelines for the evaluation of immune therapy activity in solid tumors: immune-related response criteria. *Clinical Cancer Research*.

